# Treatment Patterns and Characteristics of Dialysis Facilities Randomly Assigned to the Medicare End-Stage Renal Disease Treatment Choices Model

**DOI:** 10.1001/jamanetworkopen.2022.25516

**Published:** 2022-08-05

**Authors:** Adam S. Wilk, Kelsey M. Drewry, Rebecca Zhang, Stephen O. Pastan, Rebecca Thorsness, Amal N. Trivedi, Rachel E. Patzer

**Affiliations:** 1 Rollins School of Public Health, Emory University, Atlanta, Georgia; 2 Emory University School of Medicine, Atlanta, Georgia; 3 Emory Transplant Center, Atlanta, Georgia; 4 Brown University School of Public Health, Providence, Rhode Island; 5 Providence VA Medical Center, Providence, Rhode Island

## Abstract

**Importance:**

In 2021, Medicare launched the End-Stage Renal Disease Treatment Choices (ETC) model, which randomly assigned approximately 30% of dialysis facilities to new financial incentives to increase use of transplantation and home dialysis; these financial bonuses and penalties are calculated by comparing living-donor transplantation, transplant wait-listing, and home dialysis use in ETC-assigned facilities vs benchmarks from non-ETC–assigned (ie, control) facilities. Because model participation is randomly assigned, evaluators may attribute any downstream differences in outcomes to facility performance rather than any imbalance in baseline characteristics.

**Objective:**

To identify preintervention imbalances in dialysis facility characteristics that should be recognized in any ETC model evaluations.

**Design, Setting, and Participants:**

This cross-sectional study compared ETC-assigned and control dialysis facility characteristics in the United States from 2017 to 2018. A total of 6062 facilities were included. Data were analyzed from February 2021 to May 2022.

**Exposures:**

Assignment to the ETC model.

**Main Outcomes and Measures:**

Dialysis facilities’ preintervention transplantations and home dialysis use, facility characteristics (notably, profit and chain status), patient demographic characteristics, and community socioeconomic characteristics.

**Results:**

Among 316 927 patients, with 6 178 855 attributed patient-months, the mean (SD) age in January 2017 was 59 (11) years, and 132 462 (42%) were female. Patients in ETC-assigned facilities had 9% (0.2 [95% CI, 0.1-0.2] percentage points) lower prevalence of living donor transplantation, 12% (3.2 [95% CI, 3.0-3.3] percentage points) lower prevalence of transplantation wait-listing, and 4% (0.4 [95% CI, 0.3-0.4] percentage points) lower prevalence of peritoneal dialysis use compared with control facilities. ETC-assigned facilities were 14% (5.1 [95% CI, 0.9-9.4] percentage points) more likely than control facilities to be owned by the second largest dialysis organization. Relative to control facilities, ETC-assigned facilities also treated 34% (6.6 [95% CI, 6.5-6.7] percentage point) fewer patients with Hispanic ethnicity and were located in communities with median household incomes that were 4% ($2500; 95% CI, $500-$4500) lower on average.

**Conclusions and Relevance:**

In this study, dialysis facilities in ETC-assigned regions had lower preintervention prevalence of transplantation wait-listing, living donor transplantation, and peritoneal dialysis use, relative to control facilities. ETC-assigned and control facilities also differed with respect to other facility, patient, and community characteristics. Evaluators should account for these preintervention imbalances to minimize bias in their inferences about the model’s association with postintervention outcomes.

## Key Points

**Question:**

How are the dialysis facilities randomly assigned to Medicare’s End-Stage Renal Disease Treatment Choices (ETC) model different from control facilities?

**Findings:**

In this cross-sectional study of 6062 facilities treating 316 927 patients, patients receiving treatment at ETC-assigned facilities had lower prevalence of living-donor transplantation, transplantation wait-listing, and peritoneal dialysis use than those treated at control facilities. ETC-assigned and control facilities were also different with respect to facility ownership and patient demographic characteristics.

**Meaning:**

These findings suggest that ETC model evaluators should account for these preintervention imbalances in outcomes and other participant facility characteristics to minimize bias in their analyses of the model’s associations with outcomes.

## Teaser

This cross-sectional study assesses characteristics of dialysis facilities randomly assigned to Medicare’s End-Stage Renal Disease Treatment Choices (ETC) model in comparison with control facilities.

## Introduction

More than 780 000 US residents require kidney replacement therapy to treat kidney failure, also known as end-stage renal disease. Persons with kidney failure, most of whom undergo dialysis treatment, have poor survival and other health outcomes,^1^ and for Medicare—the predominant payer for dialysis services in the United States^1^—their health care is disproportionately costly. In 2018, individuals with kidney failure represented 1% of all Medicare beneficiaries but 7% of Medicare’s fee-for-service expenditures.^1^

As part of efforts to improve the value of this population’s care, Medicare implemented the End-Stage Renal Disease Treatment Choices (ETC) model effective January 2021.^2^ Unlike previous voluntary payment reforms for kidney care providers, ETC participation was mandatory for all dialysis facilities (and, in parallel, all nephrology practices that manage the care of patients undergoing dialysis) in 30% of US hospital referral regions (HRRs).^3^ Because Medicare assigned HRRs to ETC randomly (within census region strata),^3^ the ETC model represents the largest randomized trial of payment reform in US history and is unprecedented as a potential source of robust evidence on the impact of payment reforms on care outcomes. The model is also among the first Medicare payment models to include structural components that directly address health equity.^4,5^

The ETC model’s principal goal is to increase use of kidney transplantation and home dialysis, treatment options associated with lower costs, greater patient convenience, and (for transplantation) greater survival, compared with conventional in-center hemodialysis.^6,7,8,9,10^ Beginning July 2022, the ETC model will award escalating payment bonuses or penalties to ETC-assigned dialysis facilities (and, separately, to nephrology practices) based on (1) their prevalent patients’ home dialysis use, living donor transplantation, and transplant wait-listing, compared with control facilities in HRRs not randomly assigned to ETC, and (2) the ETC-assigned facilities’ rate of improvement in these treatment outcomes. By 2027, the model will increase facility payments by as much as 8% or reduce them by as much as 10%.^3^

Prior research has shown wide variability in access to kidney transplantation and home dialysis among dialysis facilities, attributed to combinations of patient-,^11,12,13^ neighborhood-,^14,15^ facility-,^16,17^ and health-system–level^18,19,20^ factors, which are often geographically concentrated (eg, due to racial/ethnic segregation^21^). Thus, by chance, ETC-assigned and control facilities may be different on average with respect to transplantation use, home dialysis use, and factors associated with opportunities to improve these treatment outcomes. We use national data to compare dialysis facilities randomly assigned to the ETC model and control facilities with respect to their baseline patient treatment outcomes and other facility characteristics. By identifying the nature of any preintervention differences between these facility groups, our findings could both aid evaluators in making correct inferences about the causal effect of the ETC model and anticipate potential upward or downward payment adjustments for ETC-assigned dialysis facilities based on performance relative to control facilities.

## Methods

### Data Sources

We used multiple data sources to construct a national cohort of dialysis facilities and define characteristics at facility and community levels. While important facility characteristics are available from 2020 Dialysis Facility Compare and Dialysis Facility Reports, these data sets do not capture key patient-level outcomes or clinical characteristics. We therefore merged these data with the most recent available United States Renal Data System (USRDS) data set, which we used to identify dialysis facilities treating patients during 2017 to 2018. The USRDS collects information on nearly all US patients with kidney failure from the time of dialysis start (eg, demographic and clinical characteristics, insurance) and includes information on kidney failure treatments and mortality.^1^ We linked our facility-level data to zip code tabulation area–level characteristics from the 2019 five-year American Community Survey (ACS), including population-level socioeconomic data.

This study was approved by Emory University’s Institutional Review Board. A waiver of informed consent was granted for this retrospective, deidentified study. We followed the Strengthening the Reporting of Observational Studies in Epidemiology (STROBE) reporting guidelines for observational studies.

### Study Cohort

Our cohort comprises US dialysis facilities providing treatment during 2017 to 2018. We based our sample construction on treatment patterns for prevalent patients, in alignment with ETC model payment incentives.^3^

We identified all individuals in the USRDS who received any kidney replacement therapy during 2017 to 2018, including surviving individuals who had received a living-donor kidney transplant during July 2016 or later (no dialysis treatment during 2017-2018). Of these 526 376 individuals, we excluded 7027 (1.3%) who were younger than 18 years and 96 370 (18.3%) older than 75 years, 17 555 (3.3%) for whom demographic data were not available, 13 979 (2.7%) who were residing or receiving dialysis treatment in a nursing home, and 42 568 (8.1%) who did not have traditional (fee-for-service) Medicare as a primary or secondary payer for at least 1 month; these patients are excluded from ETC model measures.^3^ Additionally, we censored patient-month–level data following dialysis treatment discontinuation, kidney function recovery, or loss to follow up (6339 patients). In parallel, from the 10 592 unique facilities that provided at least 1 dialysis treatment during 2017 to 2018, we excluded 344 facilities (3.2%) based in Maryland and US territories because they were not subject to randomization under ETC, 690 facilities (6.5%) that were Veterans Administration–affiliated or based in hospitals or transplant centers, and 123 facilities (1.2%) with missing facility identifiers. We then merged our patient and facility data at the patient-month level, assigning patient-months to the facility where the patient was treated last during the month, using patients’ treatment facility identifiers. No patients were matched to 2126 facilities (22.5% of remaining facilities). We excluded 1247 facilities (13.2%) that were low-volume (<11 attributed patient-years^3^ on average per operating year). These attribution and exclusion criteria mirrored the population denominator for ETC model performance measures.^3^ Our final analytic sample included 6062 dialysis facilities and 6 178 855 patient-months of treatment at those facilities among  316 927 unique patients. A diagram illustrating our study cohort’s construction is presented in the eFigure in the Supplement.

### Outcomes and Measures

This study compared dialysis facilities based on location in a HRR randomly assigned to the ETC model.^3^ We designated dialysis facilities ETC-assigned if their zip codes listed in Dialysis Facility Compare were located in ETC-assigned HRRs (96 of 306 HRRs nationwide^22^; 1891 facilities) and control otherwise (4171 facilities). Facilities’ treatment use outcomes and patient characteristics—aggregated to the facility level—as well as facility and community characteristics were compared. We focused on treatment use outcomes associated with ETC model incentives, including living-donor kidney transplantation, transplantation wait-listing, use of home dialysis (any, peritoneal dialysis, home hemodialysis), in-center hemodialysis, and in-center self-dialysis (all modalities).^3^ These outcomes were identified at the patient-month level using the patient’s last service received during each month. Patient mortality was defined as deaths (all-cause) per 1000 attributed patient-years.

Facility-level characteristics were obtained from Dialysis Facility Compare or Dialysis Facility Reports. These included chain membership and profit status (coded as large dialysis organizations A and B [large for-profit chains], small for-profit chain, independent for-profit, or not-for-profit),^23^ peritoneal dialysis training availability, home hemodialysis training availability, and social worker–to-patient ratio—a modifiable facility characteristic associated with patient education and referral for transplantation evaluation.^24^ From USRDS data we derived facility-level average patient characteristics used in the ETC model’s risk adjustment and stratification protocols: mean age (transplant rates are age-adjusted) and percentage with Medicaid insurance (ie, dually Medicare- and Medicaid-enrolled).^3,25^ Other aggregated patient characteristics included percentages female; percentages Hispanic, non-Hispanic Asian American, non-Hispanic Black or African American, non-Hispanic White, and other race/ethnicity (American Indian or Alaska Native, Native Hawaiian or other Pacific Islander, and other [unspecified]); percentage with a body of mass index (calculated as weight in kilograms divided by height in meters squared) greater than 35; and percentage having received pre–kidney failure nephrology care. We define race as a social construct, not a biological one,^26^ and interpret race and receipt of pre–kidney failure nephrology care as proxies for unmeasured social risk factors, including those associated with access to care (eg, poverty, social position). From the ACS, we included community-level socioeconomic characteristics: urban location (vs rural), median household income, percentage below the federal poverty level, percentage who completed at least some college, and percentage unemployed.

### Statistical Analysis

Two-sided *t* tests for continuous variables and χ^2^ tests for categorical variables were used to compare ETC-assigned and control facilities’ characteristics, using 5% levels for detecting significance and applying Bonferroni corrections to account for multiple hypothesis testing.^27,28^ We compared ETC-assigned and control facilities after applying patient-month–volume weights. Patient-months were attributed to facilities based on the site of treatment during the month. For patients who received a living donor transplant during or after July 2016 and received no dialysis treatments during 2017 to 2018, all 2017 to 2018 patient-months were attributed to the last known site of outpatient dialysis treatment, following ETC attribution rules. SAS version 9.4 (SAS Institute) was used for all analyses.

## Results

Our analytic cohort included 6062 US dialysis facilities and 316 927 patients with 6 178 855 attributed patient-months. Among these patients, the mean (SD) age in January 2017 was 59 (11) years, and 132 462 (42%) were female. The mean (SD) facility-level prevalence of living donor transplant, kidney transplant wait-listing, and peritoneal dialysis treatment was 2.2% (14.8), 25.6% (43.6), and 8.8% (28.3) of attributed patient-months, respectively, and mean (SD) mortality was 132.0 (1256.2) deaths per 1000 patient-years (Table 1). Compared with control facilities, ETC-assigned facilities had 9% (0.2 [95% CI, 0.1-0.2] percentage points) lower prevalence of living donor transplantation, 12% (3.2 [95% CI, 3.0-3.3] percentage points) lower prevalence of wait-listing, and 4% lower prevalence of peritoneal dialysis treatment use (8.6% vs 9.0%, respectively; 0.4 [95% CI 0.3-0.4] percentage points). Patient mortality was similar between ETC-assigned facilities and control facilities.

**Table 1.** Prevalent Patient Treatment and Outcomes, Overall and by ETC Region Status, January 2017 to December 2018^a^

Treatment or outcome	Mean (SD), %			ETC-assigned vs control, mean difference (95% CI), percentage points
	All facilities (N = 6062)	ETC-assigned facilities (n = 1891)	Control facilities (n = 4171)	
Living donor kidney transplant	2.2 (14.8)	2.1 (14.5)	2.3 (15.0)	−0.2 (−0.2 to −0.1)
Wait-listed for a kidney transplant	25.6 (43.6)	23.4 (42.3)	26.6 (44.2)	−3.2 (−3.3 to −3.0)
Receiving home dialysis^b^				
All types	10.9 (31.2)	10.0 (31.0)	11.1 (31.4)	−0.2 (−0.3 to −0.1)
Peritoneal dialysis^c^	8.8 (28.3)	8.6 (28.0)	9.0 (28.7)	−0.4 (−0.4 to −0.3)
Home hemodialysis	2.1 (14.3)	2.2 (14.8)	2.1 (14.2)	0.2 (0.1 to 0.2)
Self-dialyzing in-center (all modalities)	0.02 (1.4)	0.02 (1.3)	0.02 (1.5)	−0.007 (−0.01 to −0.003)
Receiving hemodialysis in-center	84.5 (36/1)	85.0 (35.7)	85.3 (35.4)	0.6 (0.5 to 0.7)
Deaths per 1000 patient-years, mean (SD)^d^	132.0 (1256.2)	129.5 (1245.1)	133.1 (1262.4)	−3.6 (−5.7 to −1.5)
Patients				
No.	68 467I	20 907	47 560	NA
Attributed patient-months, No.	6 178 855	1 923 749	4 255 106	NA

Abbreviations: ETC, End-Stage Renal Disease Treatment Choices model; NA, not applicable.

^a^ Facility-level values weighted by attributed patient-months; *t *tests were used to obtain 95% CIs, comparing variable values for ETC-assigned vs control facilities, with Bonferroni corrections for 27 comparisons applied.

^b^ Dialysis modality statistics identified using the US Renal Data System Detailed Treatment History RXHIST file.

^c^ Peritoneal dialysis statistics include continuous ambulatory peritoneal dialysis, continuous cycling peritoneal dialysis, and other peritoneal dialysis.

^d^ Patient-months for deaths per 1000 patient-years (including patient-months with missing modality information): all facilities, 6 225 263; ETC-assigned facilities, 1 937 093; and control facilities, 4 288 170.

About three-fourths (4486 [76%]) of dialysis facilities were owned by 1 of 2 large dialysis organizations, about half (3210 [54.5%]) offered peritoneal dialysis training, and one-third (1855 [31.5%]) offered home hemodialysis training (Table 2). Relative to control facilities, ETC-assigned facilities were 14% (5.2 [95% CI, 0.9-9.4] percentage points) more likely to be owned by large dialysis organization B and 37% (2.0 [95% CI, 0.2-3.9] percentage points) less likely to be for-profit and independent. ETC-assigned and control facilities were similarly likely to offer peritoneal dialysis training and home hemodialysis training and had similar social worker staffing.

**Table 2.** Characteristics of US Dialysis Facilities and Their Attributed Prevalent Patients, Overall and by ETC Region Status, January 2017 to December 2018^a^

Variable	Mean (SD) %			ETC-assigned vs control, mean difference (95% CI) , percentage point
	All facilities (N = 6062)	ETC-assigned facilities (n = 1891)	Control facilities (n = 4171)	
**Facility characteristics**				
Ownership and profit status, No. (%)				
Large for-profit dialysis organization				
A	2270 (38.5)	663 (36.2)	1607 (39.6)	−3.4 (−7.7 to 0.9)
B	2216 (37.6)	754 (41.2)	1462 (36.0)	5.1 (0.9 to 9.4)
Small for-profit chain,	612 (10.4)	218 (11.9)	394 (9.7)	2.2 (−0.5 to 4.9)
Independent, for-profit	289 (4.9)	64 (3.5)	225 (5.5)	−2.0 (−3.9 to −0.2)
Not-for-profit	505 (8.6)	133 (7.3)	372 (9.2)	−1.9 (−4.4 to 0.6)
Peritoneal dialysis training available, No. (%)	3210 (54.5)	946 (51.6)	2264 (55.8)	−4.1 (−8.5 to 0.2)
Home hemodialysis training available, No. (%)	1855 (31.5)	564 (30.8)	1291 (31.8)	−1.0 (−5.1 to 3.1)
Social workers per 100 patients, No.	2.0 (1.2)	2.0 (1.2)	1.9 (1.1)	0.1 (−0.1 to 0.2)
**Characteristics of attributed kidney failure patients**				
Patient age, y	58.3 (11.4)	58.1 (11.4)	58.4 (11.3)	−0.3 (−0.3 to −0.3)
Female	41.7 (49.3)	41.7 (49.3)	41.7 (49.3)	0.1 (0.0 to 0.2)
Hispanic	17.6 (38.1)	13.0 (33.6)	19.6 (39.7)	−6.6 (−6.7 to −6.5)
Non-Hispanic Asian American	4.2 (20.1)	3.1 (17.3)	4.7 (21.1)	−1.6 (−1.6 to −1.5)
Non-Hispanic Black or African American	38.7 (48.7)	41.6 (49.3)	37.4 (48.4)	4.1 (4.0 to 4.3)
Non-Hispanic White	36.7 (48.2)	38.7 (48.7)	35.8 (48.0)	2.9 (2.8 to 3.0)
Other race/ethnicity	2.8 (16.4)	3.6 (18.6)	2.4 (15.3)	1.2 (1.2 to 1.3)
Medicaid insurance	27.0 (44.4)	26.8 (44.3)	27.1 (44.4)	−0.2 (−0.3 to −0.1)
BMI >35	27.3 (44.5)	27.8 (44.8)	27.1 (44.4)	0.7 (0.6 to 0.8)
Pre–kidney failure nephrology care	57.6 (49.4)	59.1 (49.2)	56.9 (49.5)	2.1 (1.9 to 2.2)
Community characteristics				
Urban (vs rural), No. (%)	5977 (99.1)	1867 (99.3)	4110 (99.0)	0.3 (−0.4 to 1.1)
Median household income, thousands of US dollars, mean (SD)	60.2 (23.8)	58.4 (22.7)	60.9 (24.2)	−2.5 (−4.5 to –0.5)
Residents with household income <100% FPL	11.9 (7.9)	12.1 (7.9)	11.8 (7.8)	0.3 (−0.4 to 1.0)
Residents with some college education	27.3 (13.6)	26.8 (13.3)	27.6 (13.7)	−0.8 (−2.0 to 0.4)
Unemployed	3.6 (1.7)	3.7 (1.8)	3.6 (1.7)	0.0 (−0.1 to 0.2)
Attributed patient-months, No.	6 178 855	1 923 749	4 255 106	NA

Abbreviations: ETC, End-Stage Renal Disease Treatment Choices model; BMI, body mass index (calculated as weight in kilograms divided by height in meters squared); NA, not applicable.

^a^ Facility-level average values weighted by attributed patient-months; 95% CIs obtained from *t* tests for continuous variables and χ^2^ tests for categorical variables, comparing variable values for ETC-assigned vs control facilities, with Bonferroni corrections for 27 comparisons applied. Data sources and sample size: attributed patient characteristics (age and race and ethnicity dispersions) and ratio of social workers to patients derived from Dialysis Facility Reports (1891 ETC-assigned facilities; 4171 control facilities); available dialysis modalities and facility ownership and profit status derived from Dialysis Facility Compare (linked sample: 1832 ETC-assigned facilities; 4060 control facilities); community characteristics (urbanicity, median household income, poverty, unemployment, and educational attainment) derived from the American Community Survey (1880 ETC-assigned facilities, 4153 control facilities).

Relative to patients in control facilities, ETC-assigned facilities’ patients were 34% (6.6 [95% CI, 6.5 to 6.7] percentage points) less likely to be Hispanic (13.0% vs 19.6%), 34% (1.6 [95% CI, 1.5 to 1.6] percentage points) less likely to be non-Hispanic Asian American (3.1% vs 4.7%), 11% (4.1 [95% CI, 4.0 to 4.3] percentage points) more likely to be non-Hispanic Black or African American (41.6% vs 37.4%), and 50% (1.2 [95% CI, 1.1 to 1.2] percentage points) more likely to have other racial and ethnic identity (3.6% vs 2.4%). Patients in ETC-assigned facilities were also 0.3 (95% CI, 0.3-0.3) years younger, on average, than patients in control facilities. Percentages of patients with Medicaid insurance were not meaningfully different (26.8% vs 27.1%; −0.2 [95% CI, −0.1 to −0.3] percentage points). ETC-assigned facilities’ patients had a 3% (0.7 [95% CI, 0.6 to 0.8] percentage points) greater likelihood of having a body mass index greater than 35 (27.8% vs 27.1%) and a 4% (2.1 [95% CI, 1.9 to 2.2] percentage points) greater likelihood of receiving pre–kidney failure nephrology care (59.1% vs 56.9%). Relative to control facilities’ communities, the communities of ETC-assigned facilities had lower median household incomes ($58 400 vs $60 900; mean difference, $2500 [95% CI, $500 to $4500]) but were otherwise similar.

## Discussion

Medicare’s ETC model, which aims to increase transplantation and home dialysis use among adults with kidney failure, represents the largest randomized trial of financial incentives for health care facilities in US history. We examined the ETC model’s randomization procedure and found that preintervention (2017 to 2018) prevalence of living-donor transplantation, transplant wait-listing, and peritoneal dialysis treatment use were 9% (2.1% vs 2.3%), 12% (23.4% vs 26.6%), and 4% (8.6% vs 9.0%) lower, respectively, among ETC-assigned dialysis facilities’ patients compared with patients at control facilities. These findings have implications for evaluations of the ETC model’s impact and for dialysis facility payments under ETC.

We observed imbalances in baseline characteristics between ETC-assigned and control dialysis facilities that evaluators must account for statistically to the extent possible to minimize bias in their inferences about the ETC model’s impact. In the context of randomized trial studies, researchers commonly assume that the treated and control groups are, on average, similar with respect to any characteristics that may affect outcomes. However, in any one experiment, important participant characteristics, including baseline outcomes and key covariates, may be unbalanced and lead to biased inferences. If ETC evaluators do not adequately account for the baseline differences we observed, they could underestimate the model’s impact: for example, they could conclude that the ETC model was associated with a small decrease in transplant wait-listing when ETC-assigned facilities may have actually increased their patients’ placement on transplant waiting lists as much as or more than control facilities. The potential implications of such biased inferences could include the refinement, restructure, or preemptive termination of the model, despite its (theoretical) benefits.

The literature is equivocal about whether adjusting for such baseline imbalances is appropriate in evaluating randomized trial results. Methodologists agree that imbalances will affect the results of the trial, yet some argue that any adjustment can impair causal inference by inducing imbalance in unmeasured factors.^29,30^ Others recommend addressing imbalances using different study designs and analytic approaches, some of which could be deployed with future large-scale randomized interventions and payment models under Medicare. These alternatives include prespecifying variables potentially correlated with outcomes and controlling for them using various techniques,^31,32^ using cluster-randomized crossover trial designs,^33^ or rerandomizing assignment.^34^ Recognizing that the ETC model is under way, the literature offers 2 methodological approaches that preserve its randomization procedure’s experimental nature while accounting for preintervention imbalances. The first is to report—alongside the main, unadjusted findings—the results of sensitivity analyses that adjust for differences between ETC-assigned and control facilities in (1) baseline values of the outcome measures or (2) select covariates potentially correlated with the outcomes and with ETC assignment, including community-level median household income.^35^ The second is to stratify ETC-assigned and control facilities by these characteristics and report outcomes within strata. The latter approach aligns with how Medicare has adjusted for differences in social risk factors between incentivized and benchmark facilities under other Centers for Medicare & Medicaid (CMS) Innovation payment models.^36,37,38^ Medicare could also consider abandoning the ETC model’s experimental nature in favor of quasi-experimental evaluation strategies (eg, difference-in-differences, comparing changes in outcomes over time between ETC-assigned and control facilities).

Because the ETC model adjusts participating dialysis facilities’ payments downward if they have low use of living donor transplantation, transplant wait-listing, and home dialysis use vs benchmark levels among control facilities, our findings indicate that the average ETC-assigned facility is disproportionately likely to receive a financial penalty. The magnitude of any penalty (or bonus) changes at the 30th, 50th, 75th, and 90th percentiles of control facility performance; thus, the impact of the baseline differences we observed on payments to ETC-assigned facilities would be concentrated among facilities with performance near these thresholds. The ETC model does not randomize patients to facilities (or facilities to communities); therefore, having a robust risk adjustment protocol remains essential to adhere to the CMS Innovation Center’s updated strategic objectives^5^ and avoid penalizing facilities that serve disproportionately socially vulnerable populations. Under the ETC model’s risk adjustment protocol, CMS will age-adjust transplantation rates and stratify facilities by the proportion of patients dually enrolled in Medicare and Medicaid or who receive Medicare’s low-income subsidy.^3,25^ This protocol may account for some of the average differences between ETC-assigned and control facilities that we observed in patient-, facility-, and community-level characteristics. Future studies of facility-level associations between social risk factors and low transplant and home dialysis use among ETC-assigned facilities could inform further revisions to CMS’s protocol as needed.^39^

Additionally, as with any randomized trial, the ETC model intervention may elicit heterogeneous treatment responses. We found that ETC-assigned facilities were more likely to be for-profit and chain-affiliated facilities (vs not-for-profit or independent facilities) relative to control facilities. These results—alongside our main findings of less transplantation and home dialysis use in ETC-assigned facilities—are consistent with evidence that, controlling for other factors, patients in for-profit, chain-operated dialysis facilities have lower likelihoods of receiving a transplantation, being wait-listed for a transplant, and using home dialysis compared with patients in non-for-profit facilities.^23,40,41,42^ However, the large magnitude of the ETC model’s payment adjustments (ranging from 8% increases to 10% reductions by 2027) may drive for-profit dialysis facilities to respond proactively to the ETC model’s new incentives and increase their patients’ transplantation and home dialysis use disproportionately. In particular, large dialysis organizations may draw on their experiences with Medicare’s Comprehensive ESRD Care Initiative (2015-2021) to guide performance improvements in their ETC-assigned facilities^43,44,45^ (eg, by enhancing interorganizational communication,^46^ deploying new patient education programming^47,48,49^). Consequently, relative to not-for-profit facilities, for-profit facilities may be more likely to experience payment adjustments based on improvement in their patients’ home dialysis use and transplant wait-listing status, rather than based on achievement relative to control facility benchmarks.^3^ It will be important to monitor how facilities respond differently to ETC model incentives by profit and chain status.

### Limitations

Our study has limitations. First, our data did not support replicating exactly all ETC model rules in constructing our analytic sample. Notably, we could not exclude patients with dementia or who used hospice services. Since we found ETC-assigned facilities’ patients were younger on average, our main results may be attenuated by any associated bias. Second, while the ETC model randomizes both dialysis facilities and managing nephrology practices in selected HRRs to new financial incentives,^3^ this study focuses on dialysis facilities alone. Third, although more recent data are not yet available, our findings for facilities treating patients during 2017 to 2018 may not fully generalize to the contemporary population of dialysis facilities. In particular, we did not examine trends over time in treatment outcomes for ETC-assigned and control facilities; any differences in these trends (through 2020) could affect how our findings translate to the current era. Additionally, future research should examine whether ETC-assigned and control facilities may be different with respect to other factors unobservable in our data but potentially associated with treatment outcomes, such as patient social support and community-level nephrologist supply.

## Conclusions

In this study of dialysis facilities randomized into the ETC model, ETC-assigned facilities and control facilities were different with respect to key treatment outcomes. The lower baseline prevalence of transplantation and home dialysis use among ETC-assigned facilities (vs control facilities) may lead ETC model evaluators to underestimate the ETC model’s effects if they assume that, because of randomization, treated and control dialysis facilities are alike with respect to characteristics that may affect treatment outcomes. Strategic use of sensitivity and stratified analyses that account for these baseline differences can help evaluators make correct inferences about the model’s impact.

## Notes

Supplement. **eFigure.** Study Cohort Construction
