# Supplementary material for: Treatment Patterns and Characteristics of Dialysis Facilities Randomly Assigned to the Medicare End-Stage Renal Disease Treatment Choices Model
Source: JAMA Netw Open. 2022 Aug 5;5(8):e2225516. doi: 10.1001/jamanetworkopen.2022.25516 (PMC9356315; doi:10.1001/jamanetworkopen.2022.25516)
Supplement: Supplement. — eFigure. Study Cohort Construction [file jamanetwopen-e2225516-s001.pdf]

Supplemental Online Content

Wilk AS, Drewry KM, Zhang R, et al. Treatment patterns and characteristics of dialysis facilities randomly assigned to the Medicare End-Stage Renal Disease Treatment Choices model. *JAMA Netw Open.* 2022;5(8):e2225516. doi:10.1001/jamanetworkopen.2022.25516

eFigure. Study Cohort Construction

This supplemental material has been provided by the authors to give readers additional information about their work.

© 2022 Wilk AS et al. *JAMA Network Open.*

eFigure. Study Cohort Construction

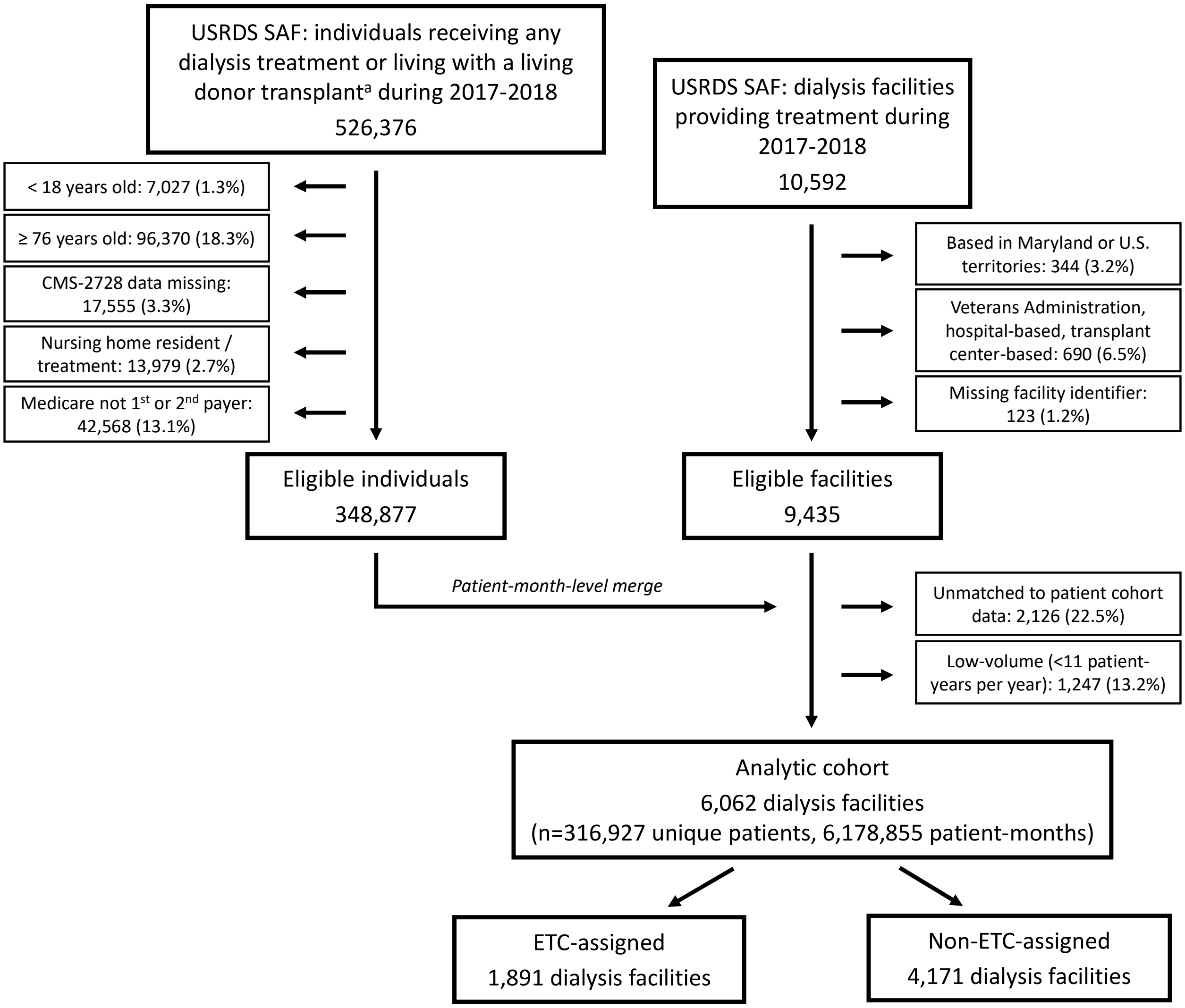

[a] Includes individuals whose living donor transplant event took place during or after July 2016

© 2022 Wilk AS et al. *JAMA Network Open.*
